# Exploring the experiences of imposter phenomenon among Chinese ICU nurses: a qualitative study

**DOI:** 10.3389/fpsyg.2026.1772552

**Published:** 2026-03-25

**Authors:** Fucheng Zhu, Xueling Fang, Shaofang Luo, Rufang Deng, Mingzhu Wang, Yanhuan Li

**Affiliations:** 1 Department of Nursing, Zhuhai Campus of Zunyi Medical University, Guangdong, China; 2 Department of Nursing, School of Medicine, The Sixth Affiliated Hospital of South China University of Technology (Nanhai District People's Hospital of Foshan), Foshan, China

**Keywords:** imposter phenomenon, intensive care unit, nurses, professional experiences, qualitative research, qualitative study

## Abstract

**Background:**

The high intensity and complexity of ICU nursing subjects practitioners to significantly greater occupational stress and burnout risks than those working in general wards. Burnout directly impacts nursing quality and is closely linked to the imposter phenomenon. This latter factor is a significant contributor to burnout and may exacerbate its severity. Therefore, it is crucial to gain a thorough understanding of how the imposter phenomenon develops and manifests among ICU nurses in order to stabilize their career trajectories and enhance their professional well-being.

**Aim:**

This study aimed to explore ICU nurses’ experiences of imposter phenomenon, providing a reference for the development of relevant future intervention strategies.

**Study design:**

We employed a phenomenological study design and collected data. Using purposive sampling combined with snowball sampling, 15 ICU nurses from a tertiary general hospital were selected for semi-structured interviews from June to July 2025. The Colaizzi seven-step analysis method was utilized, and the NVivo 12.0 software was used for data processing.

**Findings:**

This study identified three main themes and eight sub-themes: multiple causative factors (insufficient professional knowledge and experience, comparison with colleagues, authority effect, overloaded responsibilities, collective culture), negative emotional experiences, and diverse coping strategies (social support, cognitive restructuring, self-regulation).

**Conclusion:**

The etiology of impostor syndrome among ICU nurses is complex and multifaceted, yet targeted support systems remain inadequate. Nursing managers should prioritize this issue and establish a systematic, multifaceted support framework to promote the occupational well-being of nurses.

## 1 Introduction

### 1.1 Background

The Imposter Phenomenon (IP) was first proposed by Clance in 1978 during their research on high-achieving women. It describes an internal psychological experience where individuals, after achieving success, tend to attribute it to external factors—such as luck or others’ assistance—rather than their own abilities (Clance and Imes, 1978; Jiang et al., 2022). This is accompanied by persistent self-doubt and a denial of self-worth. As research deepened, scholars discovered this phenomenon transcends specific genders or occupational groups, manifesting across diverse age, racial, and professional backgrounds. Its causes are complex and multifaceted, involving both individual personality traits (such as perfectionism and neuroticism) and being closely linked to organizational culture and social environments (Cokley et al., 2024; Bravata et al., 2020; Gottlieb et al., 2020; Walker and Saklofske, 2023). This cognitive bias not only distorts individuals’ objective self-assessment but also constitutes a deep-seated psychological distress. It significantly increases the risk of mental health issues like anxiety and depression and can even hinder the formation of professional identity and the healthy development of one’s career (Halgas et al., 2024; Al Lawati et al., 2025).

In the medical field, where professional skills and immediate judgment are paramount, the impact of imposter phenomenon is particularly pronounced (Siddiqui et al., 2024). Research indicates that approximately 62% of healthcare professionals worldwide—including physicians, medical students, and nurses—report experiencing imposter phenomenon to varying degrees. This phenomenon shows a significant positive correlation with academic burnout, occupational burnout, and turnover intentions (Salari et al., 2025; Franchi and Russell-Sewell, 2023; Jefferson et al., 2024). Across multiple medical disciplines, the incidence of imposter phenomenon remains high in nursing, with some studies indicating rates exceeding 70% (Edwards-Maddox, 2023). This psychological state not only erodes nurses’ professional confidence but also directly impacts their clinical judgment and work engagement, thereby exacerbating occupational burnout (Peng et al., 2022). Nursing burnout has long been a focus of attention, with consequences extending beyond individual physical and mental exhaustion to include potential declines in care quality, increased medication errors, and frequent patient safety incidents (Li et al., 2024). Research indicates that a global nursing shortage of 4.5 million by 2030, making the mental health and professional stability of nursing teams a critical priority in global public health (Yu et al., 2025). Therefore, in-depth exploration of the imposter phenomenon among nursing staff—particularly those in high-risk environments—holds significant theoretical and practical implications.

The Intensive Care Unit (ICU) is characterized by high intensity, high pressure, and high uncertainty (Jia et al., 2025). As one of the most challenging workplaces in a hospital, ICU nurses must not only master complex medical techniques and operate sophisticated equipment but also make rapid judgments and responses in rapidly changing clinical situations. Simultaneously, they endure prolonged exposure to patients’ life-and-death situations, confront highly emotional demands from families, and bear immense pressure from communication and coordination within multidisciplinary teams (Quesada-Puga et al., 2024; Ramírez-Elvira et al., 2021). This demanding professional environment and the high expectations associated with their role mean that even highly experienced and skilled ICU nurses may frequently experience feelings of inadequacy. Despite receiving praise from colleagues, promotions, or achieving clinical successes, they may attribute these accomplishments to external factors, leading to profound professional distress (Jang et al., 2025). Research indicates that burnout rates among ICU nurses exceed 90% (Li et al., 2023; Li et al., 2023; Papazian et al., 2023), highlighting the urgent need to address this group’s psychological well-being and the underlying drivers behind it.

Attribution theory offers a crucial theoretical perspective for understanding the imposter phenomenon (Luo, 2009). It posits that individuals typically attribute their successes and failures to dimensions such as personal competence, effort level, task difficulty, and luck. Those inclined to deny their own competence tend to attribute success to unstable, uncontrollable external factors (such as chance opportunities or others’ assistance), while attributing failure to internal, stable, and difficult-to-change deficiencies (such as lack of ability). This cognitive pattern profoundly influences individuals’ emotional experiences and subsequent behavioral motivations; it not only perpetuates a vicious cycle of self-doubt and anxiety but also hinders individuals from drawing professional confidence and growth momentum from positive feedback. Consequently, it weakens their adaptability and resilience in high-pressure environments (Peng et al., 2025).

Despite growing attention to the imposter phenomenon in recent years, existing research has primarily been grounded in Western cultural contexts. Studies examining nurses’ experiences with imposter phenomenon within intensive care settings from an East Asian cultural perspective, such as China, remain insufficient. This research gap hinders our deep understanding of the genuine emotions of East Asian ICU nurses during their professional development. Therefore, exploring the professional experiences of East Asian ICU nurses not only deepens our understanding of the universality and cultural specificity of impostor phenomenon but also provides crucial evidence for developing culturally appropriate psychological intervention strategies.

### 1.2 Aim

This study aimed to explore the authentic experiences of intensive care nurses of imposter phenomenon and establish a reference framework for the mitigation of such tendencies within the context of the ICU.

## 2 Methods

This study adhered to the Standards for Reporting Qualitative Research to ensure transparent and comprehensive reporting of the methods and findings (Doyle et al., 2020).

### 2.1 Setting and sample

The purposive sampling and snowball sampling were adopted, selecting 15 nurses working in the general intensive care unit (ICU) of a tertiary hospital in Foshan City during June to July 2025. Inclusion criteria for the study were as follows: ① currently practicing nurses holding a valid nursing license; ② ≥ 3 months’ experience in critical care unite; ③ Informed consent and voluntary participation in the study. Exclusion criteria included the following: ① Inability to continue participation due to illness, personal leave, external training, or study; ② Experience of a major negative life event within the preceding month.

### 2.2 Recruitment

First, we contacted the nurses’ manager to acquire authorization, after which the manager distributed a recruiting announcement via the WeChat (a free messaging and calling app widely used in China) group to preliminarily identify persons interested in the study topic. The description of an IP was then described in detail to these potential subjects to guarantee their complete understanding of the role’s specific consequences, followed by self-assessment. Based on this, we did the final review and confirmation using predetermined inclusion and exclusion criteria.

### 2.3 Data collection tools and methods

#### 2.3.1 Determining the structure of the interview

Based on the literature review and expert discussion, the research team developed a set of interview questions and conducted pre-interviews with two nurses. The interview questions were revised in accordance with the pre-interview results. The main interview is shown in Table 1.

**Table 1:** The interview guide.

Interview questions
Have you experienced imposter phenomenon regarding your capabilities? Could you describe the incident or person involved? How did you feel at the time? In your view, what scenarios might trigger imposter phenomenon? What factors do you believe contribute to such an imposter phenomenon? How did you manage or respond after experiencing imposter phenomenon? Regarding the topic of imposter phenomenon among ICU nurses, do you have any further points to add?

#### 2.3.2 Data collection method

In this study, the phenomenological research method was used to conduct face-to-face semi-structured interviews with the interviewees. Each interview lasted 25–30 min and involved 15 nurses. Throughout the interview, the researchers followed the outline and employed interview tactics such as summary, repetition, and response to allow the respondents to speak freely and express their actual views. During the interview, the researchers maintained a neutral attitude, adjusted the questioning strategy based on the interviewees’ responses, carefully observed and recorded the interviewees’ facial expressions, tone of voice, and emotional changes, and finally asked the interviewees to add anything as a closing statement to ensure the accuracy of the interview.

### 2.4 Data analysis

Within 24 h of the end of each interview, the interviewer transcribed the interview recordings into written materials on time, marked the pronunciation, intonation, and pauses of the contents in detail, checked them against the interview records one by one, and kept the original recordings for double review to ensure transcription accuracy. The materials were stored and managed in the NVIVO 12.0 software. Colaizzi’s seven-step data analysis (Liu, 2019) was used to analyze the data. (1) Researchers meticulously recorded and carefully reviewed interview data from 15 participants, considering its overall significance; (2) Meaningful statements regarding causes, feelings, coping strategies, and other aspects related to IP experiences were extracted; (3) Key statements were summarized and distilled from each transcript; (4) Common characteristics or concepts among meaningful statements were identified, forming 3 themes and 8 subthemes; (5) These themes were closely linked to the research phenomenon and described in detail; (6) All findings were integrated to describe the reasons, feelings, and coping strategies associated with the IP experience, providing a detailed account of the fundamental structure underpinning this phenomenon; (7) All participants were asked to provide their contact information to ensure results were returned to them via WeChat or email for content verification, Ensuring research findings accurately reflect their experiences and perspectives. Where discrepancies arose, the research team collectively deliberated to establish definitive themes.

### 2.5 Ethics statement

This study had been approved by the Ethics Committee of the Sixth Affiliated Hospital of South China University of Technology (September 8, 2025, NYKY-2025-251-01). All participants provided written informed consent and voluntarily participated in the research.

### 2.6 Rigour

The research team comprised five supervisors and one researcher. The supervisors included three clinical nursing specialists in critical care, one nationally certified Level II psychological counsellor with qualitative research experience, and one nursing master’s graduate possessing a background in qualitative research. The researcher is a current postgraduate student who has undergone systematic training in qualitative research methods and possesses proficient interview techniques. Prior to formal interviews, the team conducted pre-interviews to optimize the research process. First, the researcher and a supervisor repeatedly listened to the recordings and reviewed the full transcripts to ensure the transcribed text was accurate, complete, and unbiased. Second, the researcher and supervisor independently coded all interview transcripts and analyzed them to generate an initial coding list. In cases of disagreement, consensus was reached through discussion within the research team. Throughout data collection and analysis, we actively considered the role of researcher reflection while suspending personal experiences and assumptions to minimize researcher influence on the research process.

## 3 Findings

In this study, 15 interviews were conducted. The sample was predominantly female and represented a wide age range. The basic information of the nurses is shown in Table 2. The qualitative thematic analysis of the data resulted in three main themes: multiple causative factors, negative emotional experiences, and diverse coping strategies, as illustrated in Table 3. The themes and sub-themes are described in detail below.

**Table 2:** Demographics of participating nurses.

No.	Gender	Age	Education level	Professional title	Working experience (year)
N1	Female	29	Bachelor’s Degree	Nurse	7
N2	Female	31	Bachelor’s Degree	Senior Nurse	10
N3	Female	41	Bachelor’s Degree	Senior Nurse	15
N4	Female	26	Bachelor’s Degree	Nurse	6
N5	Female	27	Bachelor’s Degree	Nurse	2
N6	Female	29	Bachelor’s Degree	Nurse	6
N7	Female	24	Diploma	Nurse	3
N8	Female	42	Bachelor’s Degree	Deputy Director of Nursing	21
N9	Male	22	Diploma	Nurse	1
N10	Female	36	Bachelor’s Degree	Senior Nurse	17
N11	Female	30	Bachelor’s Degree	Nurse	8
N12	Male	25	Bachelor’s Degree	Nurse 2	2
N13	Female	23	Diploma	Nurse 1	1
N14	Male	31	Bachelor’s Degree	Senior Nurse	10
N15	Female	33	Bachelor’s Degree	Nurse	11

**Table 3:** Themes and subthemes.

Themes	Subthemes
Multiple causative factors	Insufficient professional knowledge experience, Comparison with colleagues, Authority effect Overloaded responsibilities, Collective culture
Negative emotional experiences	
Diverse coping strategies	Social support, Cognitive restructuring, Self-regulation

### 3.1 Theme 1: Multiple causes

This multiple attribution model identifies the underlying causes of IP among ICU nurses over their employment. It not only illustrates the substantial impact of the ICU work environment on nurses’ psychological states, but it also emphasizes the internal tensions and psychological problems nurses experience as they strive for professional excellence within unique cultural value frameworks.

#### 3.1.1 Insufficient professional knowledge and experience

As medicine is a constantly evolving field and nursing itself is highly practice-oriented, tacit knowledge gained via practice frequently outweighs standard textbook knowledge in the ICU. This experience, which cannot be gained overnight, frequently leads to nurses’ self-doubt and self-criticism.

*“Last Sunday I was managing that Continuous Renal Replacement Therapy (CRRT) patient. I've no idea why the CRRT machine suddenly alarmed—the replacement fluid drained completely, letting in loads of air. I still don't know how to handle it properly, so I just panicked and sought help from the senior nurse. I feel like I've managed numerous CRRT patients before, yet my competence remains poor.” (N13)*

*“Take our CPR machine, for instance. If a doctor orders immediate resuscitation but you're unfamiliar with its operation, you won't know how to proceed. After all this time, having to consult senior staff makes you feel passive. That's when self-doubt creeps in.” (N5)*

*“It might be when a patient's condition deteriorates and I fail to notice it promptly, only for someone else to spot it. That's when I start questioning my competence – wondering if I'm cut out for the ICU.” (N7)*

#### 3.1.2 Comparing with colleagues

While comparison may spur motivation for self-improvement in certain contexts, irrational comparisons can undermine an individual’s sense of self-efficacy, fostering doubt and negativity about one’s capabilities (Feng et al., 2025).

*“When other colleagues seem to be working independently while I still make minor errors, I start doubting myself. I wonder if I'm genuinely less capable than others or if this profession is truly suited to me. Such thoughts inevitably arise, particularly when comparing myself to colleagues - that feeling becomes especially pronounced.” (N12)*

*“Seeing others handle tasks with such ease makes me feel rather flustered, like I'm somehow failing.” (N7)*

*“For instance, when I joined the ICU alongside some new colleagues, others proved to be quick learners with a high capacity for work and a brisk pace. They could not only complete their own tasks but also assist others, which placed considerable pressure on me and led me to doubt my own abilities.” (N13)*

#### 3.1.3 Authority effect

As a symbol of advanced medical technology within hospitals, the ICU maintains a rigid hierarchical structure where superiors’ advice and endorsement may at times become the primary measure of individual worth, even evolving into an oppressive “gold standard” (Wanjia and Xia, 2023).

*“For instance, when you feel you've performed well or given your utmost effort, yet still fail to gain leadership approval, it can be disheartening and lead to imposter phenomenon.” (N14)*

*“When senior colleagues criticize me, I question myself. I feel I've done adequately, yet they dismiss it—it's distressing. Subsequently, anxiety sets in, permeating my entire shift, leading to self-denial.” (N9)*

*“When others don't acknowledge you, it's like when you're doing ward rounds with a doctor (perhaps a senior consultant) and they might say, “That's not how it's done; you don't understand.” That's when you start to doubt yourself a bit.” (N8)*

#### 3.1.4 Overburdening of responsibility

Given the critical condition of patients and the intricate complexity of duties within the ICU, nurses may sometimes internalize any minor or even unavoidable outcome as a significant personal failure.

*“Because we deal with patients directly, for instance, during your shift, a patient might be progressing favorably, but then suddenly their condition deteriorates, leading to an unfavorable outcome. My first instinct is to reflect on myself: where might I have fallen short in caring for this patient? Or failed to provide adequate support? Why did their condition develop in this way? That's how I tend to think.” (N3)*

*“When the (medical) director criticizes your nursing practices, it feels like it lands squarely on your shoulders. As the nursing manager, I must shoulder that responsibility—managing them (the nurses) or guiding them towards better practices. It makes me feel I haven't done enough in that regard.” (N8)*

*“For instance, when resuscitation efforts fail, or when a patient doesn't recover despite intervention, you inevitably question your own actions.” (N13)*

#### 3.1.5 Collective culture

In China, Confucian culture (centered on familial ethics, upholding the value orientation whereby the individual is subordinate to the family collective and integrates the self into the greater community) and collectivist culture have been passed down for many years, which have also always been followed by the Chinese people. Deeply steeped in that culture and operating within the unique environment of the ICU, Chinese ICU nurses frequently demonstrate a pronounced sense of teamwork and collective ethos following successful patient resuscitation. They tend to attribute personal achievements to collaborative teamwork and the support of others.

*“After successfully resuscitating a patient, I feel it's the collective effort of the whole team. In the ICU, we are one team. Without their (the doctors') medical orders and treatment plans, the patient might not have recovered so quickly, but without our nursing care, the patient might not have recovered so quickly either. So I feel it's a collective achievement; I rarely think of myself.” (N7)*

*“I consider it the team's achievement in the ICU, because sometimes when you haven't noticed a patient's condition, the team will spot it for you and immediately call you to handle it together. Teamwork is crucial in the ICU; going it alone is actually very difficult. You don't think of yourself first.” (N9)*

*“Truthfully, much of our department's work relies on the team to function; it's not something one person can accomplish alone. So I feel much of the sense of achievement comes from the team. Personally, I don't feel particularly accomplished, but as a member of the ICU team, I feel successful and share in a sense of collective honor.” (N13)*

### 3.2 Theme 2: Experiences of negative emotions

When confronted with the imposter phenomenon, all interviewed nurses reported experiencing adverse emotions.

*“It leaves you feeling utterly miserable, quite oppressed.” (N3)*

*“The initial feeling is undoubtedly profound sadness, followed by intense anxiety.” (N7)*

*“There's a sense of shame, especially when you've been repeatedly reminded and still make such mistakes. Then comes the anxiety —after all, being tagged by name in a group chat for all to see…” (N12)*

*“It's definitely unpleasant, even anxiety-inducing. After the anxiety, how can one perform better to regain that confidence?” (N14)*

### 3.3 Theme 3: Diverse coping strategies

Faced with the imposter phenomenon, ICU nurses have created a positive coping resource system that includes measures like social support, cognitive restructuring, and self-regulation. This process reveals practitioners’ psychological plasticity and represents their transition from passive endurance to actively generating professional meaning—a shift in agency.

#### 3.3.1 Social support

Respondents in this study indicated they employ various methods to vent negative emotions, such as seeking support from family, friends, and colleagues. This not only alleviates internal pressure but also sustains emotional bonds.

*“Sometimes I'll vent to my family—complain about how knackered I am at work today, or about new patients I've taken on, just those sorts of things (not very pleasant matters).” (N1)*

*“I'd be feeling utterly despondent, head hanging low, convinced I'm no longer cut out for this line of work. But a single word of praise from my mentor would lift my spirits, making me realize I can still improve and do better.” (N7)*

*“With such immense work pressure, you absolutely need someone to confide in about all that inner bitterness and exhaustion; otherwise, you'd go mad. That's why we have a close-knit group of friends who meet up every few days to let off steam.” (N3)*

#### 3.3.2 Cognitive restructuring

Proactively adjusting negative interpretations and evaluations of events, employing a positive and optimistic perspective to console and encourage oneself, thereby restoring psychological equilibrium (Jiang et al., 2023).

*“Like when those professional titles were announced, seeing someone who joined at the same time already promoted to full senior level. I'd find myself a reason —he was an undergraduate, had a higher foundational qualification, and the promotion criteria back then weren't as strict as they are now. Sometimes I'd console myself like that.” (N8)*

*“Actually, sometimes lacking confidence just means you feel inadequate in some area. Anyway, I think it's perfectly normal to lack confidence. You just need to improve where you're lacking.” (N11)*

*“My approach is to focus on solving the problem rather than dwelling on the negative.” (N15)*

*“I try to stay positive, reassuring myself that it's alright, that I'm the best, and not to let other things get to me.” (N6)*

#### 3.3.3 Self-regulation

Some interviewees indicated that to avoid causing unnecessary worry for family and others, they preferred to process negative emotions internally.

*“Sometimes when I'm under immense work pressure or feeling particularly low, I hesitate to share it with my family because I fear causing them concern. It's that tendency to share only the good news and not the troubles—I just self-regulate.” (N5)*

*“I sleep it off and forget what happened yesterday.” (N7)*

*“I keep a diary. If I see something upsetting, I write it down when I get home, and that's the end of it.” (N6)*

## 4 Discussion

### 4.1 Addressing the multifaceted causes of imposter phenomenon among ICU nurses through stratified management and guidance

The imposter phenomenon encompasses individual, interpersonal, and environmental factors (Caizhi and Tang, 2025). This study indicates that ICU nurses’ IP does not stem from a single factor but is instead shaped by a combination of factors, including insufficient professional knowledge and experience, comparisons with colleagues, the authority effect, responsibility overload, and collective culture. The sustained pressure arising from the ICU as a high-stress, high-uncertainty work environment significantly impacts nurses’ psychological state (Jang et al., 2025), undermining their self-efficacy and inducing doubt about their capabilities. The predominant triggers of IP differ markedly across nurses’ career stages. Junior nurses, despite possessing theoretical foundations or clinical training, encounter a reality shock when confronted with the high-intensity, complex ICU setting during transitional phases (Ryan et al., 2025; Kang et al., 2025). Their lack of experience causes them to make horizontal comparisons with peers rather than vertical comparisons with themselves. This blind comparison, which disregards individual developmental differences, ultimately crystallizes into a fixed mindset of self-denial —the belief that one is incompetent and unsuited to the profession. For senior nurses in stable career phases, IP is closely linked to responsibility overload and the authority effect. Professional ethics demand that nurses prioritize patient-centered care and respect for life (Wang, 2001). As the final line of defense for patients’ lives, decisions and actions taken by nurses in the ICU can significantly influence patient outcomes. However, the inherent uncertainty of medicine itself can lead to adverse patient outcomes. According to attribution theory, the internal attribution tendency leads nurses to attribute failures to their own inadequacies. The ICU environment further reinforces this cognitive bias, intensifying the imposter phenomenon. Moreover, authoritative recognition from superiors (such as nursing managers) is often a key metric by which senior nurses gauge their self-worth, playing a pivotal role in how they perceive their own value (Lao et al., 2025). When nurses exert maximum effort yet receive no acknowledgement or affirmation, their professional identity is directly impacted, creating the illusion that they are inadequate or unfit for ICU practice. This study reveals that collective culture exerts a dual influence on ICU nurses’ tendency to deny their own capabilities: it simultaneously fosters a sense of belonging while extinguishing feelings of individual accomplishment. On the one hand, collective culture provides vital psychological support and safety nets for nurses under high-pressure conditions. However, a strong collective culture can blur and extinguish individual accomplishment, leading nurses to attribute success to external factors. This may be related to the emphasis on humility and collectivism in East Asian organizational cultures (Zhang et al., 2024; Choi and Lee, 2025), which constitutes a significant divergence from studies on self-competence negation in Western individualistic contexts. Consequently, nursing managers should implement precise, tiered management strategies. Junior nurses should be given the opportunity to accelerate their skill proficiency and psychological adaptation through simulation exercises (Fang et al., 2022) and one-to-one mentoring schemes (Li et al., 2021), transforming their perception of inexperience into a sense of rapid growth. For senior nurses, a system of fact-based feedback and recognition should be established. When raising issues, superiors should provide specific improvement plans rather than simply negating them. At the same time, individual unique contributions should be explicitly acknowledged in team commendations to prevent the extinction of personal achievement.

### 4.2 Recognizing the dual nature of ICU nurses’ imposter phenomenon experiences

This study demonstrates that the primary emotional experience caused by IP for ICU nurses is a severe negative emotional state that includes anxiety, despair, and humiliation. This aligns with previous research (Salari et al., 2025; Wang and Li, 2023), which confirms that IP is a significant risk factor for mental health issues and occupational burnout among healthcare professionals. People with imposter syndrome often have a tendency to doubt their own abilities and attribute their successes to external factors, rather than their own merits and efforts. This cognitive pattern significantly diminishes their sense of intrinsic accomplishment, leading to negative self-evaluation. The emotional states that accompany this syndrome, including anxiety and guilt, continuously deplete psychological resources, leading to prolonged emotional exhaustion. It is noteworthy that emotional exhaustion and diminished personal accomplishment represent two interrelated core dimensions within the theory of occupational burnout (Edwards-Maddox, 2023). At the same time, errors occur when nurses engage in catastrophic thinking due to limited experience or capability, which can rapidly escalate from ‘I made a mistake’ to ‘I harmed the patient,’ ‘I will be punished,’ and ‘I am fundamentally unsuited to nursing.’ The guilt felt towards patients, coupled with fear for their professional future, significantly exacerbates emotional exhaustion, creating precursors to occupational burnout too. However, some interviewees also perceived IP as having positive aspects, reflecting similarities with the research of Tewfik (2022). While this study and Tewfik’s research differ in perspective, both challenge the entrenched belief that negative self-perception is always harmful, acknowledging its potential constructive role in specific circumstances. Moderate imposter phenomenon can serve as intrinsic drivers of reflective practice and continuous learning, forming an essential ‘negation of negation’ process in professional development. Consequently, nursing managers must distinguish between destructive self-denial and constructive self-reflection. The former represents a psychological risk requiring intervention, while the latter, moderate, fact-based self-doubt, functions as intrinsic motivation for professional prudence and reflective practice (El Boghdady and Ewalds-Kvist, 2025). Timely identification and management are essential through measures such as establishing psychological relief rooms and conducting group counseling sessions (Fainstad et al., 2022), providing secure channels for emotional release. On the other hand, nurses should be encouraged to cultivate a growth mindset (Xue et al., 2025), viewing IP experiences as opportunities to learn rather than as verdicts of failure. Setting small, specific, and achievable work objectives enables nurses to build confidence by achieving incremental successes in their roles.

### 4.3 Establishing a multifaceted support system to provide diverse support for ICU nurses

Despite facing IP distress, this study found that ICU nurses demonstrated positive coping resilience, including seeking social support, cognitive restructuring, and self-regulation. This aligns with the findings of Ikhlaq et al. (2023) and Xu et al. (2024), who indicated that individuals actively utilize all available resources to compensate for psychological resource depletion. Social support, cognitive restructuring, and self-regulation are pivotal psychological resources and cognitive-emotional regulation strategies that have been extensively researched for their role in alleviating negative emotions and promoting psychological adaptation (Zhang et al., 2025; Kelly et al., 2022). Specifically, social support functions primarily by providing emotional comfort and practical help, which helps individuals to reduce emotional distress, boost their confidence in coping with adverse events, and increase their reserves of resources, thus maintaining their physical and mental well-being. Cognitive restructuring involves guiding individuals to replace original negative interpretations with more adaptive, reality-based assessments. It alters their cognitive evaluation of stressful events, effectively reducing subsequent physiological arousal and subjective distress (Jiang et al., 2023). Meanwhile, self-regulation enables individuals to proactively employ strategies such as cognitive reappraisal and situational adjustment. This enables them to maintain goal-directed behavior when confronted with negative stimuli, avoiding being dominated by immediate emotions and thereby preserving the stability of cognitive and behavioral functioning. However, these coping mechanisms in this study, whether confiding in family and friends, receiving praise from colleagues, or self-reassurance, predominantly exhibit individualized and informal characteristics. It reflects the current absence of systematic, institutionalized psychological support systems at the organizational level. Consequently, at the individual level, nursing managers should conduct cognitive behavioral therapy workshops (Moore et al., 2024) to teach nurses to identify common negative thought patterns such as perfectionism and excessive responsibility. This helps nurses distinguish between controllable and uncontrollable factors, focusing on targeted interventions for attribution restructuring, which yields more lasting effects in alleviating IP. Establish convenient, confidential psychological service channels, such as setting up hospital-based counseling hotlines and anonymous appointment systems, with dedicated psychologists providing regular consultations. At the team level, implement peer support education and a mentorship program where each newly hired or ICU-rotating nurse is paired with a trained senior nurse as a support partner (Hu et al., 2025). This pairing not only guides clinical skills but also provides regular emotional support and early identification of psychological stress indicators. At the systemic level, foster an organizational culture that embraces imperfection and establish multi-departmental collaborative support mechanisms (Li et al., 2023). Nursing managers should lead by example, sharing lessons learned from mistakes and normalizing clinical uncertainties to alleviate nurses’ overloaded responsibilities. Collaborate with the Nursing Department, labor union, and psychiatric department to establish a Nurse Mental Health Committee. This committee should regularly survey nurses’ psychological needs, dynamically update support measures, and disseminate relevant concepts through hospital bulletin boards, official accounts, and other channels to enhance awareness among all staff.

### 4.4 Strengths and limitations

The study examines the specific manifestations of this psychological phenomenon within China’s high-pressure social environment and the context of East Asian collectivist culture, offering a crucial perspective for comprehensively understanding this phenomenon. However, it only looked at one hospital, which may limit its generalizability.

### 4.5 Recommendations

The factors that contribute to the imposter phenomenon among ICU nurses are numerous. Nursing managers should pay special attention to the root reasons and take proactive steps to help nurses better adapt to the demands of their professional development. In the future, research may be expanded in the following areas: first, to include nursing populations from different regions and hospitals of varying tiers, thereby increasing the study’s representativeness and generalizability; and second, to conduct longitudinal tracking surveys to investigate the trajectory of nurses’ imposter phenomenon.

## 5 Conclusion

This study employs a qualitative research approach to explore the impostor phenomenon experienced by Chinese ICU nurses and their associated experiences. Findings reveal that the causes of imposter phenomenon among Chinese ICU nurses are diverse and complex, suggesting that understanding and managing this issue should extend beyond the individual psychological level. Instead, it requires examination and intervention from multiple dimensions, including the individual, interpersonal, environmental, and cultural perspectives. However, current strategies for addressing this phenomenon lack systematic and standardized protocols. Therefore, nursing managers should prioritize this issue and actively promote the establishment of institutionalized psychological support systems to more effectively assist nurses in coping with and alleviating imposter phenomenon.

## Data Availability

The original contributions presented in the study are included in the article/supplementary material, further inquiries can be directed to the corresponding author.
